# Target languages, types of activities, engagement, and effectiveness of extramural language learning

**DOI:** 10.1371/journal.pone.0253431

**Published:** 2021-06-28

**Authors:** Ruofei Zhang, Di Zou, Gary Cheng, Haoran Xie, Fu Lee Wang, Oliver Tat Sheung Au

**Affiliations:** 1 Department of Mathematics and Information Technology, The Education University of Hong Kong, Hong Kong SAR, China; 2 Department of English Language Education, The Education University of Hong Kong, Hong Kong SAR, China; 3 Department of Computing and Decision Sciences, Lingnan University, Hong Kong, Hong Kong SAR, China; 4 School of Science and Technology, The Open University of Hong Kong, Hong Kong SAR, China; Nanjing University of Information Science and Technology, CHINA

## Abstract

Since Sundqvist introduced the term “extramural English” in 2009, empirical research on extramural language learning has continued to expand. However, the expanding empirical research has yet yielded incommensurate review studies. To present a timely picture of the field of extramural language learning, this study conducts a review of 33 relevant articles retrieved from Scopus and Web of Science databases. The results showed the five types of target languages frequently investigated in this field (i.e., English, German, French, Chinese, and Japanese) and seven main types of extramural learning activities (i.e., playing digital games, watching videos, reading, listening to audios, having technology-enhanced socialisation, having face-to-face socialisation, and writing compositions). People’s engagement in extramural language learning was overall high, especially listening to audios and playing digital games, mediated by the relationship between the difficulty of the activities and people’s target language proficiency levels, gender, and the interactive environment. Extramural language learning was overall effective for language development and enhancing affective states in language learning. The effectiveness may be influenced by the involvement of language inputs and outputs and the amount of engagement time. Implications for practitioners were suggested concerning encouraging digital gameplay, emphasising formal language instruction, and creating positive interactive environments for extramural language learning.

## 1. Introduction

In pedagogical experiences, teachers have been widely aware of the comprehensive implementation of extramural language learning (hereinafter, ELL) among their students and its possible effectiveness for language development [1]. ELL refers to the learner-initiated activities beyond educational institutions that occur without an intention to learn a language but end with language educational effectiveness [2, 3]. People can develop language knowledge and skills through their engagement in leisure-time entertainment or recreation, such as watching movies, playing digital games, and having chit-chats with families and friends in the target language [1]. Along with the broadening awareness of the implementation and possible usefulness of ELL, increasingly more researchers have been interested in this language learning approach. Since Sundqvist introduced the term “extramural English” in 2009 [3], empirical research on ELL has been expanding, spanning various aspects of language learning, from a wide range of perspectives [2, 3].

So far, the escalating empirical research of ELL has yet yielded incommensurate review studies in this field. To our best knowledge, few reviews have been conducted of the previous empirical studies on ELL except [4]. [4] focused on multiplayer online role-playing gaming as a type of ELL activity and reviewed the relevant publications from 2012 to 2018, identifying its overall effectiveness and attributing the effectiveness to the socio-cultural and collaborative nature of the gaming experience. However, people might have engaged in many other types of ELL activities than multiplayer online role-playing gaming and found them effective for various language learning aspects [1, 5]. Only when people’s engagement and outcomes of all types of ELL activities have been examined can a comprehensive picture be attained of this language learning approach.

Additionally, most researches related to ELL focused on English as a second language as the target, such as [1–4], ignoring the potential of this learning approach to the development of other language types, such as Chinese as a second language and Japanese as the first language. However, language types may have impact on the outcomes and engagement of ELL. To explore the possible usefulness of ELL for developing other types of target languages than English as a second language, a review of related research articles with foci on the target languages may be helpful.

Moreover, studies published after 2018 might have yet been reviewed. As research on ELL has continued to expand for the past years [3], an overview of the latest developments and trends in the field of ELL may be timely, suggesting implications and recommendations for future implementation and investigation.

To fill the gaps, this research aims to conduct a review of previous empirical research on ELL with foci on the types of target languages, the types of ELL activities and research findings concerning people’s engagement in ELL activities and the effectiveness of ELL. The following questions guide this review:

1. What were the target languages in the previous studies of ELL?
2. What types of ELL activities were investigated?
3. How frequently did people engage in ELL?
4. Were ELL effective?
5. What factors may influence people’s engagement in ELL?
6. What factors may influence the effectiveness of ELL?

## 2. Literature review

Researchers regarded ELL as close to the concept of out-of-class language learning [3], which refer to the language learning activities that have no direct relationship to educational institutions and that learners conduct in a self-directed way out of interest [6]. Despite its similarity to ELL in terms of learners’ purpose and the relationship with schooling, out-of-class language learning takes place based on learners’ explicit consciousness of its educational purposes, while ELL usually occurs without such consciousness [3]. Another term close to ELL is extracurricular language learning [5]. It is similar to ELL in terms of the non-instructional nature and educational effectiveness [7]. However, extracurricular language learning activities are part of schoolwork, organised and evaluated by formal language teachers, while ELL is initiated and undertaken entirely by learners themselves without any intervention or assessment by schools or teachers [8].

Previous researchers have analysed the effectiveness of ELL based on diversified theories, among which the motivation theory was frequently applied [2, 3]. It contends that learners with higher motivation tend to devote more effort to learning activities and achieve better learning outcomes [9]. [2] argued that ELL activities were more motivating than conventional ones because they were of more attractive and entertaining content, such as digital games, movies, and comics. People engaged in ELL activities purely out of pleasure. Thus, they were likely to engage in ELL with high motivation and achieve satisfying language learning outcomes. Autonomy theory can also be used to explain the effectiveness of ELL [3]. It contends that learners with higher control over their learning process were more likely to achieve academic success [10]. [3] argued that learners selected, arranged, and undertook ELL activities completely at their own pace, likely to have high control over the entire ELL process, so they might achieve enhanced language learning efficiency.

## 3. Method

We conducted this review based on a three-step method: search, selection, and data analysis, following previous review studies in the field [11–13]. The articles were searched with “English” as the language, “all year” as time-span, and “article” for the required document type. Our databases were Web of Science Core Collection and Scopus that were frequently applied in previous review studies [12, 14]. We applied three groups of keywords with AND operators between them. One group was used to search the research on extramural learning, identified in the relevant literature [2, 3, 5]. They are “extramural” or “out-of-school exposure.” The second group of keywords was used to search for educational research, identified in previous review studies in this field [12, 13, 15]. They are “teach” or “learn” or “educat.” The third group of keywords was used to search for research on language, which was identified among the most used languages worldwide [16]. They are “language” or “English” or “Chinese” or “Russian” or “Japanese” or “German” or “Spanish” or “Hindi” or “Portuguese.”

**Fig 1:**
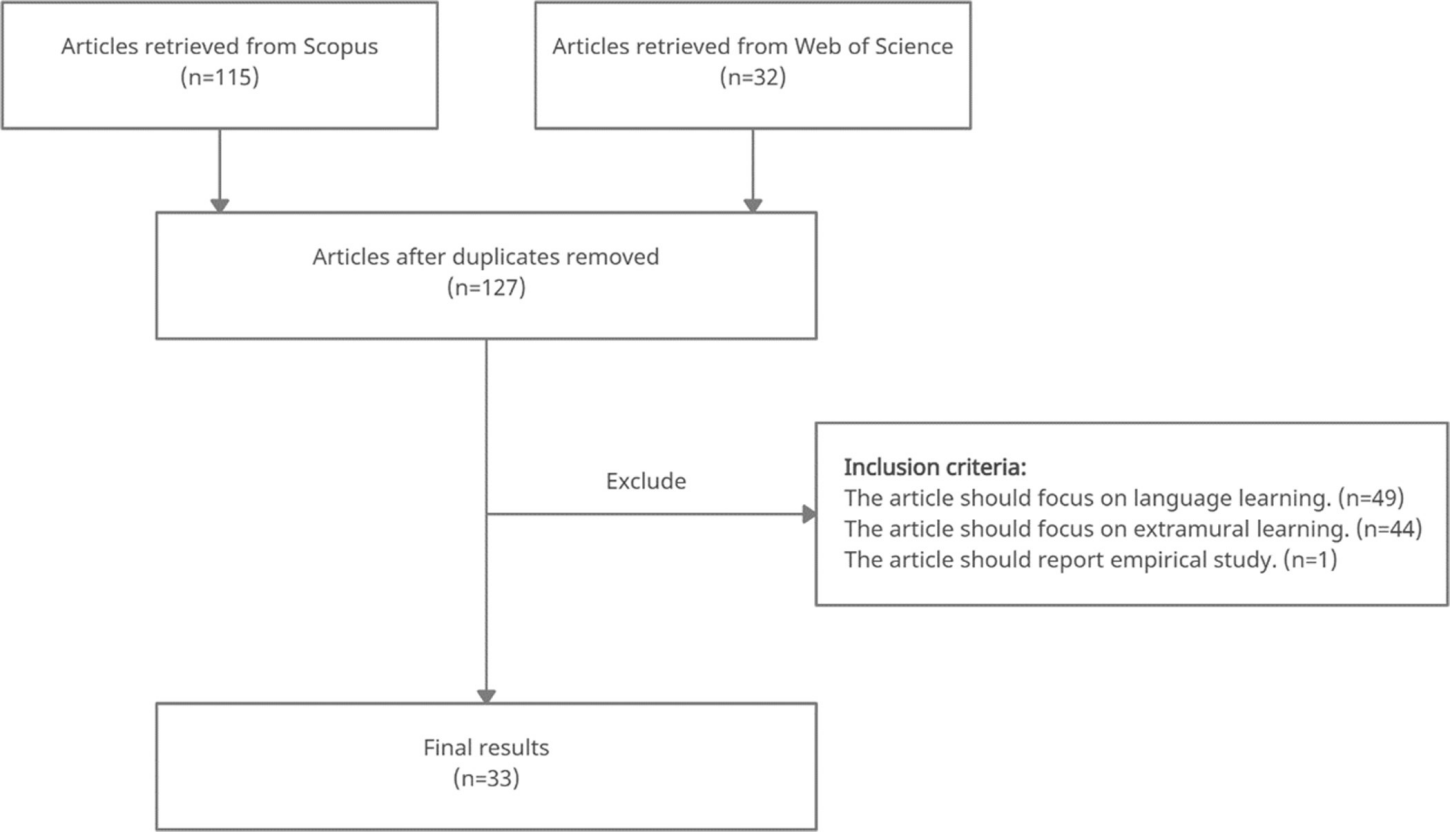
Process of data collection.

The search was conducted on March 9^th^, 2021. One hundred fifteen articles were retrieved from Scopus, and 32 from Web of Science Core Collection. Among the total 147 articles, 20 are duplicates and removed. To ensure the relevance of the reviewed articles, the remaining 127 articles were screened by the titles, abstracts, and full texts based on three inclusion criteria. First, the article should focus on language learning. This criterion excluded 49 articles. Second, the article should focus on extramural learning. This criterion excluded 44 articles. Third, the article should report an empirical study. This criterion excluded one article. Thirty-three articles were finalised in the selection, as shown in Table 1. Fig 1 illustrates the process of data collection, following PRISMA 2009 flow diagram.

**Table 1:** Reviewed articles.

Journal titles	Articles
*Mediterranean Journal of Social Sciences*	[17]
*Asia Pacific Journal of Education*	[18]
*Journal of Teaching in International Business*	[19]
*Research Papers in Education*	[20]
*Journal of Computer Assisted Learning*	[21]
*Online Submission*	[22]
*Studies in Second Language Learning and Teaching*	[23]
*Bilingualism-Language and Cognition*	[24]
*Applied Linguistics*	[25]
*Calico Journal*	[26, 27]
*British Journal of Educational Technology*	[28–30]
*Porta Linguarum*: *Revista Internacional de Didáctica de las Lenguas Extranjeras*	[31]
*Australasian Journal of Educational Technology*	[32]
*Language Learning & Technology*	[33, 34]
*Computer Assisted Language Learning*	[35]
*ReCALL*	[36–38]
*System*	[39, 40]
*Edulanguage*	[41]
*Revista Española de Lingüística Aplicada/Spanish Journal of Applied Linguistics*	[42]
*Language Learning*	[43–45]
*Apples*: *Journal of Applied Language Studies*	[46]
*ITL-International Journal of Applied Linguistics*	[47]
*Asian EFL Journal Research Articles*	[48]
*CSL 2009*	[49]

The 33 articles were analysed from four aspects.

1. Target languages. This category concerns the types of language that the subjects learned through ELL as reported in the reviewed article, including Chinese, English, French, etc.
2. Type of ELL activities. This category concerns the leisure-time activities that the subjects had for ELL, including watching movies, playing video games, reading books, etc. [3].
3. Research findings concerning people’s engagement in ELL. This category concerns the frequency and the amount of time the subjects devoted to different types of ELL activities, as reported in the reviewed article
4. Research findings concerning the effectiveness of ELL. This category concerns the usefulness of ELL activities for language learning and involves two codes, following previous reviews in this field [15]. One concerns the target aspects of language learning, including academic and affective aspects. The other code concerns the reported effects of ELL activities, falling into positive results, neutral results, negative results, and mixed results.

To begin the data analysis, the authors browsed the abstracts of the 33 articles to grab the big picture. Subsequently, we analysed five articles together for the development of the coding scheme based on the research questions. Once an agreement had been reached, we analysed the remaining articles individually based on the coding scheme and compared our coding results. The inter-rater reliability was satisfactory (Pearson’s *r* = 0.96), and the remaining differences were resolved via discussion.

## 4. Results

This section presented the results of this review from the perspectives of the types of target languages, the types of ELL activities, people’s engagement in ELL, and the effectiveness of ELL. S1 Appendix presents the summary of the results.

### 4.1. Types of target languages

**Fig 2:**
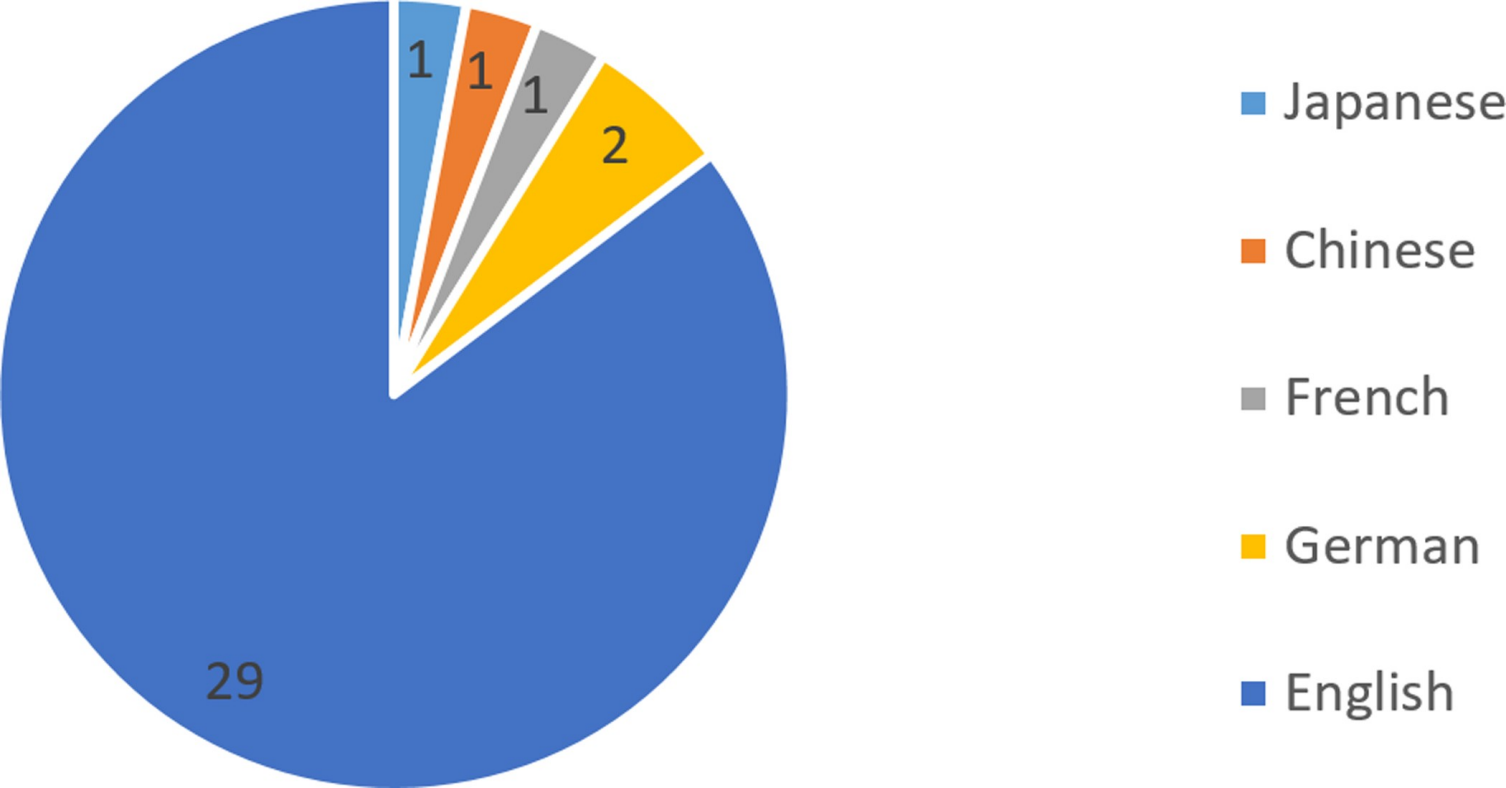
Types of the target languages.

ELL has been investigated as an approach to the development of five types of languages, as illustrated in Fig 2. English was investigated most frequently in 29 studies (88%), followed by German in two studies (6%). French, Chinese and Japanese were respectively investigated in one study (3%). All these languages were learned as the second language. [44] investigated both English and French, so the sum is bigger than 33.

### 4.2. Types of ELL activities

**Fig 3:**
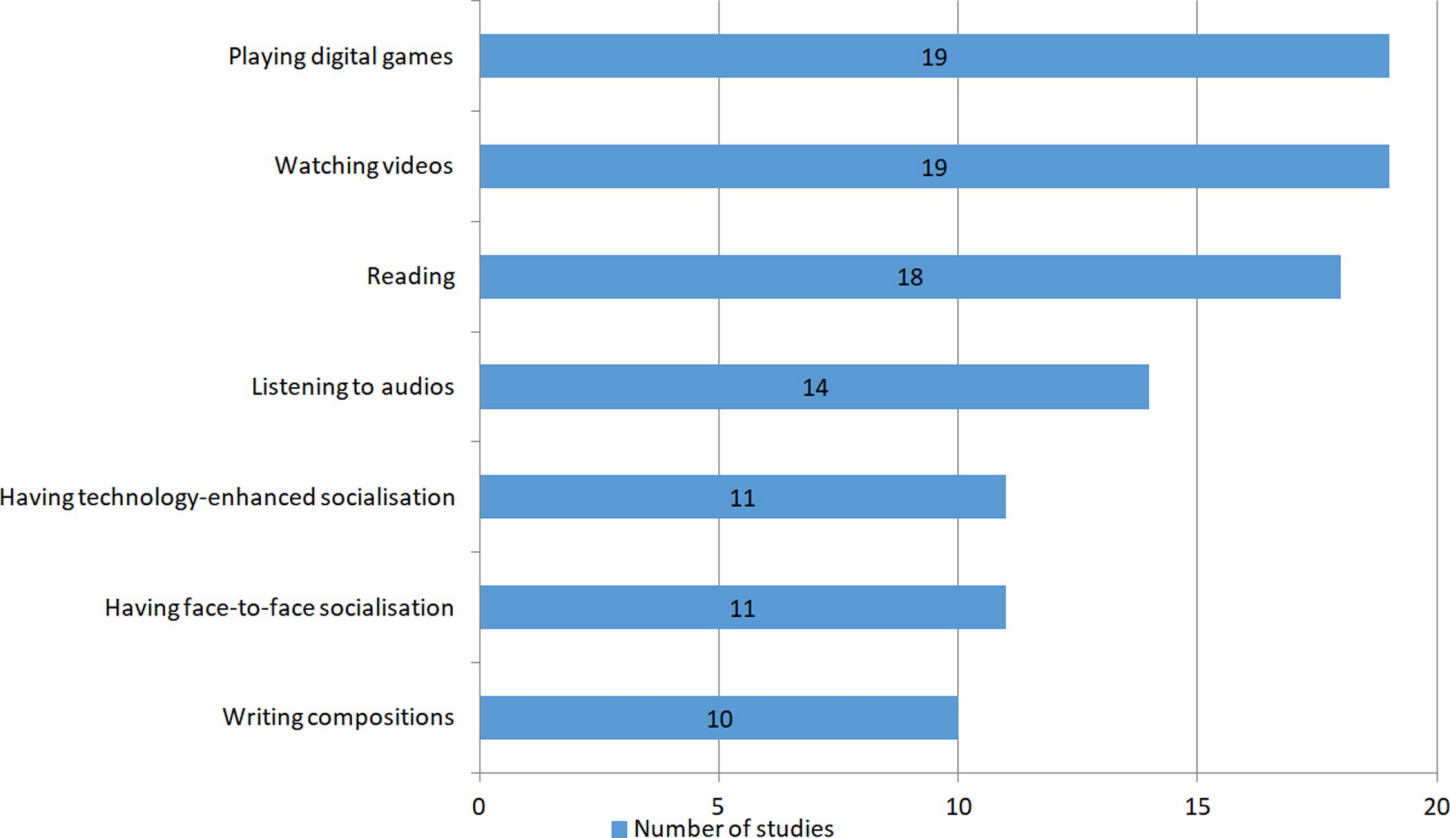
Types of ELL activities.

Fig 3 displays diversified ELL activities investigated in the literature. Playing digital games (in 19 studies) and watching videos (in 19 studies) were investigated most frequently, followed by reading (in 18 studies), listening to audios (in 14 studies), having technology-enhanced socialisation (in 11 studies) and having face-to-face socialisation (in 11 studies), and writing compositions (in 10 studies). [25, 30, 33] investigated ELL activities in general without specifying the exact types of ELL activities. The sum is bigger than 33 because most studies investigated more than one type of activity.

Increasingly valued in the field of language development [12, 50], digital gameplay has been frequently investigated as a type of ELL activity. Engaging in this activity, people had to comprehend a considerable amount of language inputs [21] to understand the rules, goals, plots, and settings of games. Also, digital gameplay usually involved competitiveness and collaboration [51], providing people with rich opportunities for interactivity in the target language with friends and strangers around the world. Interactivity as such facilitated people’s development and practice of language knowledge and skills [17]. Digital games for ELL can be online or offline [38], either single-player, multiplayer, or massively multiplayer [38, 42]. People can engage in this activity using PCs or video game consoles, such as PlayStation [26]. World of Warcraft was the game investigated most frequently in the literature [27, 34].

Watching videos in the target language can engage students in long-time exposure to language inputs through visual and audio channels [13, 15], so it was frequently investigated as a type of ELL activity. Previous research on ELL investigated diversified types of videos, including animations [18], TV series [21], spots events [32], movies [26], and YouTube video clips [29]. Video watching can be without un-subtitled [24] or subtitled in the native language [22] or foreign language [32].

Researchers were also interested in learners’ leisure reading as a type of ELL activity. Engaging in activity, people were exposed to a massive amount of vocabulary and grammar knowledge in the written form and frosted reading comprehension [47]. The reading materials investigated in the literature included books [21], comics [18], newspapers [46], journals [42], magazines [24], and manuals and product descriptions [22]. Learners can read printed materials [45], e-book [42], or websites [31].

Another activity that provided learners with language inputs was listening to audios. This activity provided people with language inputs in the spoken form and facilitated their development of listening proficiency. Researchers investigated various types of listening materials for ELL, including songs [24], lyrics [21], radios [22], and podcasts [32]. Useful tools for this activity included PCs, MP3s, and smartphones [30].

Over the years, people have been spending increasingly more time in technology-enhanced socialisation, usually using social media and communicative tools, which researchers investigated as a type of ELL activity [28, 41]. Engaging in this activity, people could search for information, read posts and comments, and keep updated about social news and friends’ status [28], exposed to extensive language inputs in authentic settings. Also, social media and communicative tools enable people to have frequent interactions with friends and strangers around the world. The interactivity could be synchronous (e.g., chatting) or asynchronous (e.g., posting and commenting), oral or textual [28, 41], in which people actively received inputs for information comprehension and produce outputs for self-expression. Technologies frequently applied in this ELL activity included Facebook, Twitter, KaKaoTalk, Line, WeChat, WhatsApp, and Skype [21, 30, 32, 46].

Having face-to-face socialisation was also investigated as a type of ELL activity. It centred on the oral, synchronous interactivity that helped people develop listening comprehension and speaking skills in authentic settings. Researchers focused on people’s use of target language in their face-to-face conversations with friends [22], family members [39], foreigners around the world [32].

Lastly, writing compositions in the target language was investigated as an ELL activity. While producing target language, people retrieved vocabulary and grammar knowledge from their long-term memory, reflect their language proficiency levels, and develop writing proficiency [15]. Researchers investigated people’s writing of emails/letters [32], messages [29], stories [22], diaries [46], and reviews [35]. People may handwrite with paper and pens or type with keyboards. Their writing outcomes can be private or public online in social sharing platforms [22] or blogs [46].

### 4.3. Research findings concerning people’s engagement in ELL activities

This review identified people’s overall high engagement in ELL in the literature [45–47]. [37] reported that their participants’ average time devoted to ELL activities was up to 57.2 hours per week. The overall high engagement may be related to the widespread language education around the world. A vast number of people had received formal language instruction. The instruction may have raised people’s awareness of the usefulness of the target language [19], improved their attitudes to the target language [46], enhanced their capabilities of and confidence in comprehending and enjoying the entertainment products in the target language [30], and, eventually increased their engagement in ELL activities [22]. Another reason for people’s overall high engagement in ELL lay in the ubiquity of entertainment products in different languages, especially English [44]. Many people grew up consuming those products, so they were accustomed to and even fond of ELL activities [44].

In addition to the high engagement in ELL in general, researchers observed people’s varying participation in different types of ELL activities [42]. Listening to audios was frequently argued as the activity in which people engaged most [23, 42, 44, 45, 47]. For particular, [42] and [26] reported that female adolescents spent the most time on this activity. The high engagement was likely because listening to audios was overall easy and relaxing, requiring little attention or application of language knowledge and skills for content comprehension [24].

Playing digital games was also reported as an ELL activity in which people frequently engaged [22, 25, 26, 52]. The competitive and interactive features of digital gameplay could excite people and sustain their attention for a long time, especially for males [37]. Researchers reported that males devoted most time and effort to this activity [42, 44, 45, 49]. [26] found that males spent around 235 minutes per week playing digital games, significantly more than females who spent around 45 minutes per week. Moreover, males’ interest in digital gameplay remained high over time, while females’ interest in this activity declined along with aging [21].

Reading may be the ELL activity in which people were involved least frequently, as reported by [44] and [47]. It was likely because reading in the target language was more demanding than other ELL activities, requiring relatively broader vocabulary and higher comprehension abilities [45, 53]. Evidence was found in [44], who reported that university students spent much more time reading in the target language than secondary students. University students had larger vocabulary sizes that enabled them to read in the target language more easily [44]. For similar reasons, adults read in the target language more frequently than teenagers [42].

People’s engagement in other types of activities was at the medium level. For example, researchers reported people’s medium-high engagement in video watching [21, 26], with female adolescents spending the most time in this activity [42]. Some people would voluntarily apply the target language in technology-enhanced socialisation [21], especially when interacting with foreign friends in familiar communities [35]. Additionally, females may devote more time to this activity than males because of their stronger sense of community and socialisation [37]. However, [35] noted that some people were reluctant to engage in technology-enhanced socialisation because they felt anxious about being judged or ridiculed by peers. [42] reported people’s medium-low engagement in face-to-face socialisation. The researcher stated that most people were willing to speak the target language with foreigners, but they usually felt uncomfortable using the language with their friends and families.

In sum, this review suggests people’s overall high engagement in ELL in general. People listened to audios and played digital games more frequently than other ELL activities, while their interest in reading in the target language may be meagre.

### 4.4. Research findings concerning the effectiveness of ELL

**Fig 4:**
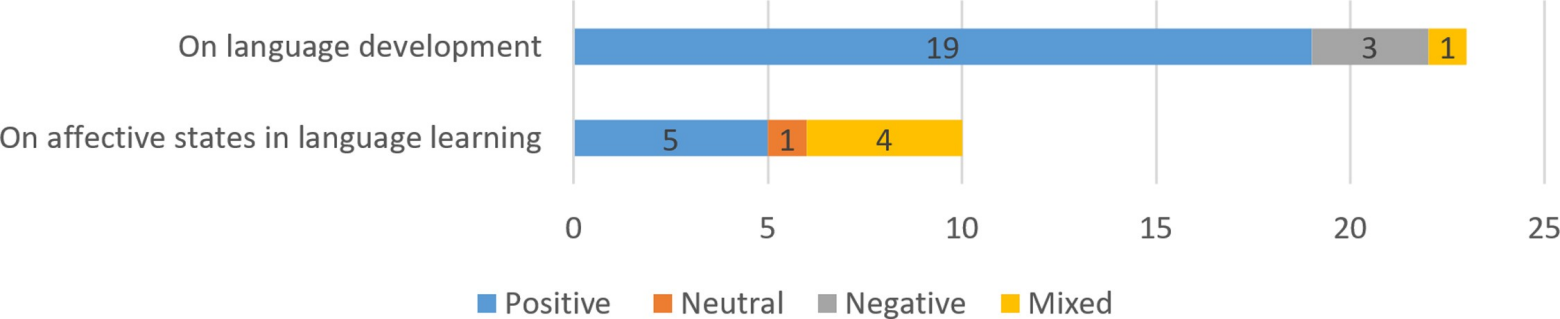
Effects of ELL.

Previous research examined the effectiveness of ELL on academic (in 23 studies, 70%) and affective aspects (in ten studies, 30%), as shown in Fig 4. As for developing language knowledge and skills, 19 studies reported positive effects of ELL (83%), three reported negative effects (13%), and one [42] reported mixed effects (4%). As for the effectiveness of ELL for enhancing people’s affective states in language learning, five studies reported positive results (50%), one [26] reported neutral results (10%), and four reported mixed results (40%).

#### 4.4.1. Effectiveness of ELL on language development

As for developing target language, most researchers reported positive results, contending that ELL could help obtain enhanced vocabulary [45, 47] and grammar knowledge [43] and writing [23], speaking [36, 49], listening [23], and reading skills [21], although no significant difference in effects was found between these aspects. The effectiveness of ELL may be explained by the considerable opportunities for contextualised language input it offered [43, 47]. In ELL, learners might have deep, long-time exposure to various aspects of language knowledge, for example, the spoken and written forms of vocabulary presented in subtitled TV programs [26, 47]. These language inputs were usually contextualised by authentic settings, such as characters, actions, and plots [21], so learners could easily comprehend them and apply them in appropriate events [33, 43, 54]. Another reason for the effectiveness of ELL concerned its affordance of an engaging and pressure-free language learning environment [19, 48]. ELL activities were fun, engaging, and self-regulated, in which learners could adjust their pace and strategies of information absorption without stress [48] and have high interest and concentration on the contents for a long time [19, 21]. Learning environment as such motivated learners and increased their language learning efficiency [26], in line with the motivation theory. The third reason for the effectiveness of ELL may lie in its interactive nature. ELL activities tended to involve people in social interaction and authentic communication [21], which was usually constrained in the conventional language classroom [27]. In the social settings, people proactively received and comprehended language inputs with high attention [39] and performed sophisticated communicative practices for self-expression [21], so they were triggered to become active users of language knowledge and skills and obtained enhanced language learning efficiency [24]. Plus, the socialisation and communication in ELL activities could be friendly and safe in which people received scaffolding, mistake correction, and warm encouragement in their language use [39].

However, ELL did not necessarily lead to language development. It could be ineffective when it reduced learners’ effort for formal language learning. Formal instruction was essential for language development, while engagement in ELL can be time-consuming, taking away time from formal learning activities (e.g., reading schoolbooks and doing homework) [24, 46]. Additionally, students who frequently engaged in ELL might undervalue the formal language instruction offered by schools, unmotivated in language lessons and school assignments, which led to their reduced language learning efficiency [25, 42]. Furthermore, ELL may not lead to satisfying language learning outcomes when it involved no language comprehension or outputs. Researchers observed that people could hardly develop language knowledge or skills by listening to audios [42, 47]. It was likely because people tended to be passive receivers of information in this activity, having little need to understand the auditory contents or produce any output [24].

#### 4.4.2. Effectiveness of ELL on affective states in language learning

ELL had overall positive effects on people’s affective states in language learning from two aspects. One concerned people’s perceptions of the target language and its cultural background. Frequently exposed to the target language in ELL activities, people tended to develop familiarity with that language [23, 36]. Meanwhile, ELL activities presented a wide range of cultural aspects, expanding learners’ knowledge of the target language’s cultural background [18, 19]. The increased familiarity and expanded knowledge may lead to people’s affective appraisal of the target language and its cultural origin [23] and help them foster a global mindset [19]. The other improved aspect was people’s motivation in formal learning and application of target language. Evidence was found that people with more ELL experiences had more enjoyment [29], motivation, and self-efficacy [37] in formal language and were more willing to use the target language [32].

However, ELL did not necessarily yield enhanced affective states in language learning. [28] reported that her participants, despite their frequent engagement in ELL activities, did not have enhanced attitudes to the target language and its cultural origin because their comprehension of ELL materials and interactivity with native speakers were highly limited by their low language proficiency and communicative skills. Also, [18] and [26] observed that some people with rich ELL experiences demonstrated little interest in formal language learning because they labelled themselves as pure consumers of entertainment products and would not automatically transfer into formal language learners. Additionally, [37] reported that engagement in ELL might increase people’s awareness of their shortcomings in target language proficiency, leading to their reduced confidence in learning and using the language.

## 5. Discussions

### 5.1. Factors that may influence peoples’ engagement in ELL

Based on the analysis of the review results, we identified three main factors that may influence people’s engagement in ELL. First concerned the relationship between the difficulty of ELL activities and people’s target language proficiency levels: people’s engagement in ELL may be high when their language proficiency level was high, and the difficulty of ELL activity was low, consistent with [17]. This is because people with higher language proficiency levels may be able to process ELL materials more easily, more efficiently, and more enjoyably, thus more motivated to engage in ELL activities [30]. People’s overall high engagement in listening to audios may be a piece of evidence. Listening to audios required relatively lower language proficiency levels than other ELL activities, so people were more willing to participate in this activity [24]. Another evidence was associated with adults’ higher engagement in ELL activities in general than the teenagers [31, 45], especially the relatively more demanding ones, such as reading [44], speaking [42], and watching un-subtitled videos [47]. Researchers explained that learners’ language proficiency level and comprehension ability usually increased with age, enabling them to conduct those complex ELL activities with increasing efficiency [31, 44].

Gender may influence people’s engagement in ELL [22, 47, 55]. [37, 46, 49] found that males were more frequently involved in ELL than females. Additionally, female and males’ preferences for diversified types of ELL activities may be different. Males may engage in playing digital games far more frequently than females [21, 45] because they were more attracted by the competitive and interactive nature of digital gameplay [37]. Females used the target language in technology-enhanced socialisation more frequently than males [42] because of their stronger sense of community and socialisation [37]. Females also spent much more time than males listening to audios [26, 49].

The third factor that may influence people’s engagement in ELL was the interactive environment. Interactivity was essential for ELL, enabling people to express themselves, share their life, and perceive their progress in language learning [17], thus attracting people’s long-term engagement in ELL [37]. Warm and familiar interactive environments of ELL, such as family settings [39], tended to motivate people to use the target language in interactivity more frequently [35]. However, if the interactive environment of ELL was emotionally unsafe or unconformable, people may be reluctant to engage in the activity [35]. Evidence was provided by [35], who reported that the students avoided using the target language in social media out of their fear of being judged and ridiculed by peers.

### 5.2. Factors that may influence the effectiveness of ELL

This study identified two main factors that place influences on the effectiveness of ELL. One was the language inputs and outputs involved therein. ELL that provided people with extensive langue inputs and encouraged them to produce outputs tended to result in positive outcomes [30, 33]. The very high effectiveness of playing digital games as ELL was a piece of evidence [22–24]. Researchers found that digital gameplay encouraged learners to perceive language information actively, understand the information in authentic settings, and apply target language knowledge and skill productively in interactivity, thereby leading to people’s language development [23, 24]. For a similar reason, engagement in face-to-face socialisation was also found effective for language devotement [39]. Counter-evidence was also reported, lying on the very low effectiveness of listening to audios for ELL as identified in the literature [24, 42, 47]. Researchers explained that people listening to audios made no language outputs, so they could hardly benefit in language development [24, 56].

The other factor regards the amount of engagement time in ELL activities. The more time people spent in ELL may result in their better language development. Evidence was found by [38], who identified the positive correlation between students’ performance in language tests and their spent time in ELL. Another piece of evidence was related to the overall better outcomes of ELL that males achieved than females [40, 45]. The better outcomes resulted from the more time and effort males tended to devote to ELL activities, especially digital game playing [38, 45]. Additionally, more amount of time in ELL usually brought about an enhanced sense of familiarity to and expanded knowledge about the target language and its cultural origin [18, 19], thereby leading to enhanced affective states in language learning [23, 36]. [33] reported a piece of evidence that students who experienced more ELL tended to feel more enjoyable and confident in language learning.

## 6. Conclusions and implications

This study presents a review of 33 papers on ELL, revealing the increasing application of this learning approach to language development. Target languages frequently investigated in this field were English, German, French, Chinese, and Japanese. The main ELL activities were playing digital games, watching videos, reading, listening to audios, having technology-enhanced socialisation, having face-to-face socialisation, and writing compositions. People’s engagement in ELL was overall high, with listening to audios and playing digital games as the activities in which people engaged most frequently and reading as the one in which they engaged least frequently. ELL was overall effective in developing language and enhancing affective states in language learning. Three factors may influence people’s engagement in ELL, specifically, the relationship between the difficulty of ELL activities and people’s target language proficiency levels, gender, and the interactive environment of ELL. The effectiveness of ELL may be moderated by the language inputs and outputs involved therein and the amount of engagement time. Based on the analysis of the results, this review may provide implications for the future practice of ELL.

### 6.1. Implications

We provide four main implications for the future implementation of ELL. Firstly, digital game playing may be recommended for ELL. This ELL activity involved extensive language inputs and outputs [23], leading to enhanced language proficiency and improved affective states in learning [24, 47]. Moreover, due to the great fun and interactive nature of this activity [37], most people enjoy playing games, willing to engage therein for a significant amount of time [21, 22]. Their high engagement in the ELL activity may lead to enhanced language learning outcomes from academic [38] and affective aspects [33]. Thus, to help students achieve satisfying ELL outcomes, teachers may recommend high-quality digital games of high educational value to their students, promoting their frequent engagement in this ELL activity. They may also employ some language teaching strategies that could trigger students’ digital game playing for ELL, such as flipped classroom [57] and mobile learning [58].

Practitioners of ELL may also attach high importance to formal language instruction. Due to the significant effectiveness of ELL, some students may undervalue the formal language instruction offered at school [25, 42]. However, ELL could not replace formal language education, as taking away too much time from formal learning activities to ELL would only lead to unsatisfying outcomes [24, 46]. Moreover, formal language instruction may increase people’s engagement in and efficiency of ELL. As shown in the literature, people who had experienced more formal language learning tended to engage in ELL activities more frequently [22, 31]. It is because formal language instruction could raise people’s awareness of the usefulness of the target language and thus motivate them to proactively get themselves involved in ELL activities [19, 46]. Plus, many ELL activities, such as reading and socialisation in the target language, were demanding, requiring relatively high language proficiency levels, communicative skills, and comprehension abilities [28, 45]. To perform these activities efficiently, students had better equip themselves with sufficient language knowledge and skills beforehand through formal instruction.

Furthermore, a positive interactive environment may be created for the implementation of ELL. This review identified the significance of the interactive environment in ELL. Positive interactive environments were emotionally safe and familiar to people [35, 39]. Environments as such may attract and sustain people’s interest in ELL, trigger their receptive and productive use of target language knowledge [24], enhance their perception of learning progress [37], and facilitate problem shooting [39], thereby increasing people’s engagement in and efficiency of ELL. However, negative interactive environments full of overwhelming peer pressure may result in people’s sense of anxiety and embarrassment, leading to their reluctance to engage in ELL [35]. Thus, to encourage students to engage in ELL and help them achieve satisfying outcomes, teachers are recommended to develop positive interactive environments for ELL. They could schedule some class time per week for students’ free discussion and sharing of their achievements and experiences in ELL. To ensure the environments to be positive for interactivity, teachers may monitor discussion and sharing and conduct interventions when necessary.

Lastly, ELL may be implemented to develop diversified types of languages. Although a majority of the review articles focused on learning English as a second language, application of ELL to develop other language types (i.e., German, French, Chinese, and Japanese) were also found in the reviewed articles and reported to be overall useful, indicating the huge potential of ELL as a language educational approach regardless of language types. Thus, practitioner of the education of various language types are encouraged to devote more time and effort to ELL activities.

### 6.2. Limitations

This study is not without its limits. First, we searched for the reviewed articles in two databases, Scopus and Web of Science. In future, researchers may consider expanding the review list by including more databases, such as AHCI, ERIC, and Google Scholar, so as to present a more comprehensive picture of the field of ELL. Second, this study focused on the types of ELL activities, people’s engagement in ELL, and the effectiveness of ELL. Future studies may consider re-reviewing the literature from other aspects, such as the target language, the subjects’ native languages and age, and sample sizes.

Finally, this review has identified people’s high engagement in ELL spanning diversified activities and suggested its apparent educational effectiveness for language development. Considering the great potential of this language learning approach, we expect further explorations of ELL from various perspectives, for example, learning behaviours in the process of ELL.

## Supporting information

S1 Appendix Investigated types of ELL activities, types of target languages, and main findings of the reviewed articles. (DOCX)

S1 Checklist Preferred Reporting Items for Systematic reviews and Meta-Analyses extension for Scoping Reviews (PRISMA-ScR) checklist. (DOCX)
