# Supplementary material for: Target languages, types of activities, engagement, and effectiveness of extramural language learning
Source: PLoS One. 2021 Jun 28;16(6):e0253431. doi: 10.1371/journal.pone.0253431 (PMC8238195; doi:10.1371/journal.pone.0253431)
Supplement: S1 Appendix — (DOCX) [file pone.0253431.s001.docx]

**S1 Appendix**. Investigated types of ELL activities, types of target languages, and main findings of the reviewed articles.

| **Authors** | **Target languages** | **ELL activities** | **Main findings** |
| --- | --- | --- | --- |
| Aksenova et al. (2015) | English | Reading (excerpts from Wikipedia) | - People’s engagement in ELL may be influenced by the involved game elements, required language proficiency levels, the interactive environment, and technology use. |
| Armour & Iida (2016) | Japanese | Watching (animations);  Reading (comics) | - ELL has positive effects on language development. - ELL has mixed effects on affective states in language learning. |
| Avello et al. (2019) | English | Reading (books, newspapers);  Watching (movies);  Speaking;  Writing | - People’s engagement in ELL may be influenced by their target language proficiency levels. - Watching videos may be the ELL activity in which people engaged most frequently. Reading may be the ELL activity in which people engaged least frequently. - ELL has positive effects on language development. |
| Azzolini et al. (2020) | English | Not specified | - ELL has positive effects on language development. |
| Brevik (2019) | English | Playing digital games;  Reading (books, news);  Watching (TV series, movies);  Listening (songs, lyrics);  Using social media | - Watching videos may be the ELL activity in which people engaged most frequently, seconded by having technology-enhanced socialisation and listening to audios. - People's engagement in ELL may be influenced by their age and gender. - ELL has positive effects on language development. |
| Coskun & Mutlu (2017) | English | Playing digital games;  Reading (books, newspapers, magazines, comics, manuals, and product descriptions);  Using social media;  Writing (emails, stories/compositions, messages, diaries, comments);  Speaking (with native speakers of the target language, with foreign tourists, with friends);  Listening (songs, lyrics, radios);  Watching (video clips, movies, TV series, animations, channels, with L1 subtitles, with L2 subtitles) | - People’s engagement in ELL was overall low. - People's engagement in ELL may be influenced by their target language proficiency levels and gender. - Listening to audios may be the ELL activity in which people engaged most frequently, seconded by playing digital games reading. Writing compositions may be the ELL activity in which people engaged least frequently. - Playing digital games may be the most effective ELL activity. |
| De Wilde et al. (2020) | English | Watching (TV series without subtitles, with L1 subtitles, with L2 subtitles);  Listening (songs);  Reading (books, magazines, comics);  Playing digital games;  Using social media;  Speaking | - ELL has negative effects on language development. - ELL has positive effects on affective states in language learning. - Playing digital games may be the most effective ELL activity, seconded by having technology-enhanced socialisation. - Listening to audios may be ineffective. |
| De Wilde & Eyckmans (2017) | English | Watching (TV series without subtitles, with L1 subtitles, with L2 subtitles);  Listening (songs);  Reading (books, magazines, comics);  Playing digital games;  Using social media;  Speaking | - Listening to audios may be the ELL activity in which people engaged most frequently. - ELL has positive effects on language development. - Playing digital games may be the most effective ELL activity. - Listening to audios may be ineffective. |
| Henry & Cliffordson (2017) | English | Not specified | - ELL has negative effects on language development. |
| Jensen (2017) | English | Playing digital games (online games, PC games, PlayStation);  Listening (songs, radios);  Reading;  Speaking;  Watching (TV series, video clips, movies);  Writing | - People’s engagement in ELL may be influenced by their gender. - Playing digital games may be the ELL activity in which people engaged most frequently, seconded by listening to audios and watching videos. - ELL has positive effects on language development. - ELL has neutral effects on affective states in language learning. |
| Lai (2019) | Chinese | Using social media | - ELL has mixed effects on affective states in language learning. |
| Lancaster (2018) | English | Reading (books, magazines);  Watching (TV series, movies);  Playing digital games;  Listening (songs) | - People’s engagement in ELL may be influenced by their target language proficiency levels. - ELL has positive effects on language development. |
| Lee & Drajat (2019) | English | Listening (songs, radios, podcasts);  Playing digital games;  Watching (TV series, movies, animations, sports events, with L2 subtitles);  Using social media;  Speaking (with native speakers of the target language, with friends, with non-native speakers of target language all over the world);  Writing (emails) | - ELL has positive effects on affective states in language learning. |
| Lee & Lee (2021) | English | Reading;  Using social media;  Playing digital games;  Watching (TV series, video clips);  Writing (messages, comments);  Speaking (with native speakers of the target language) | - ELL has positive effects on affective states in language learning. |
| Lee (2019a) | English | Not specified | - People’s engagement in ELL may be influenced by their target language proficiency levels. - The effectiveness of ELL may be influenced by the involved language inputs and outputs. |
| Lee (2019b) | English | Not specified | - ELL has positive effects on language development. - The effectiveness of ELL may be influenced by the amount of time of the engagement in ELL and the involved language inputs and outputs. |
| Lee (2019c) | English | Writing (messages, comments) | - People’s engagement in ELL may be influenced by the interactive environment. |
| Lee (2020d) | English | Speaking (with native speakers of the target language, with non-native speakers of target language all over the world);  Writing (messages) | - ELL has positive effects on language development. - ELL has positive effects on affective states in language learning. |
| Leona et al.(2021) | English | Speaking (with family, with friends)  Reading (books, magazines);  Watching (movies, TV series) | - ELL has positive effects on language development. - Having face-to-face socialisation with families may be the most effective ELL activity. - The effectiveness of ELL may be influenced by the types of ELL activities. |
| Malyndraet al. (2020) | English | Using social media | - ELL has positive effects on language development. - ELL has positive effects on affective states in language learning. |
| Muñoz (2020) | English | Watching (video clips with L1 subtitles, with L2 subtitles);  Playing digital games (single-player games, multiplayer games, massively multiplayer games);  Listening (radios, songs, podcasts);  Reading (e-books, magazines, magazines, comics, journals, newspapers, user guides);  Speaking (with native speakers of the target language, with friends, with tourists);  Writing (emails, messages);  Using social media | - Listening to audios may be the ELL activity in which people engaged most frequently, seconded by playing video games, watching videos, reading, writing compositions, and having face-to-face socialisation. - People's engagement in ELL may be influenced by their age and gender. - Listening to audios may be ineffective. - ELL has mixed effects on language development. |
| Muñoz et al. (2018) | English | Watching (movies, TV series);  Playing digital games;  Listening (songs) | - ELL has positive effects on language development. - Watching videos may be the most effective ELL activity. |
| Olsson & Sylvén (2015) | English | Using social media;  Writing (emails, messages, letters, diaries, blogs);  Reading (books, newspapers, magazines, comics);  Watching (movies);  Playing digital games | - People’s engagement in ELL was overall high. - People's engagement in ELL may be influenced by their target language proficiency levels and gender. |
| Peters (2018) | English | Listening (songs);  Reading (books, newspapers, magazines, comics);  Watching (movies, TV series, with or without subtitles) | - People’s engagement in ELL was overall high. - People's engagement in ELL may be influenced by their age and gender. - Listening to audios may be the ELL activity in which people engaged most frequently, seconded by watching videos. Reading may be the ELL activity in which people engaged least frequently. - Watching videos may be the most effective ELL activity. - Listening to audios may be ineffective. - ELL has positive effects on language development. |
| Peters et al. (2019) | French , English | Speaking (with friends, with family, with tourists);  Writing (messages) | - People’s engagement in ELL may be influenced by their target language proficiency levels, gender. - Listening to audios may be the ELL activity in which people engaged most frequently, seconded by watching videos and playing digital games. Reading may be the ELL activity in which people engaged least frequently. - Watching videos may be the most effective ELL activity. - ELL has positive effects on language development. |
| Puimège & Peters (2019) | English | Playing digital games;  Reading (books, newspapers);  Watching (video clips with or without subtitles);  Listening (songs) | - People’s engagement in ELL was overall high. - People's engagement in ELL may be influenced by their age and gender. - Listening to audios may be the ELL activity in which people engaged most frequently. Reading may be the ELL activity in which people engaged least frequently. - Watching videos and playing digital games may be the most effective ELL activities. - ELL has positive effects on language development. - The effectiveness of ELL may be influenced by the amount of time of the engagement in ELL. |
| Qasim (2021) | English | Playing digital games | - ELL has positive effects on language development. |
| Scholz & Schulze (2017) | German | Playing digital games | - ELL has positive effects on language development. |
| Scholz (2017) | German | Playing digital games | - ELL has positive effects on language development. |
| Sundqvis & Wikström (2015) | English | Playing digital games | - The effectiveness of ELL may be influenced by the amount of time of the engagement in ELL. |
| Sundqvist & Sylvén (2014) | English | Playing digital games;  Reading (books, newspapers, magazines);  Watching (movies, TV series);  Listening (songs) | - People’s engagement in ELL was overall high. - People's engagement in ELL may be influenced by their gender and the interactive environment. - ELL has mixed effects on affective states in language learning. |
| Sundqvist (2009) | English | Playing digital games;  Reading (books, newspapers, magazines);  Watching (movies, TV series);  Listening (songs) | - People’s engagement in ELL may be influenced by their gender. - ELL has positive effects on language development. - Playing digital games and reading may be the most effective ELL activities. |
| Sylvén & Sundqvist (2012) | English | Playing digital games (single-player games, multiplayer);  Reading (books, newspapers, magazines);  Watching (movies, TV series);  Listening (songs) | - Playing digital games may be the most effective ELL activity. - The effectiveness of ELL may be influenced by the amount of time of the engagement in ELL and the involved language inputs and outputs. |
