# Supplementary material for: Target languages, types of activities, engagement, and effectiveness of extramural language learning
Source: PLoS One. 2021 Jun 28;16(6):e0253431. doi: 10.1371/journal.pone.0253431 (PMC8238195; doi:10.1371/journal.pone.0253431)
Supplement: S1 Checklist — (DOCX) [file pone.0253431.s002.docx]

**Preferred Reporting Items for Systematic reviews and Meta-Analyses extension for Scoping Reviews (PRISMA-ScR) Checklist**

| **SECTION** | **ITEM** | **PRISMA-ScR CHECKLIST ITEM** | **REPORTED ON PAGE #** |
| --- | --- | --- | --- |
| **TITLE** | | | |
| Title | 1 | Types of activities, engagement, and effectiveness of extramural language learning. | 1 |
| **ABSTRACT** | | | |
| Structured summary | 2 | **BACKGROUND:** Since Sundqvist introduced the term “extramural English” in 2009, empirical research on extramural language learning has continued to expand. However, the expanding empirical research has yet yielded incommensurate review studies.  **OBJECTIVE:** This study aims to present a timely picture of the field of extramural language learning.  **DESIGN:** A review was conducted of 33 relevant articles retrieved from Scopus and Web of Science databases with foci on the types of ELL activities and research findings concerning people’s engagement in ELL activities and the effectiveness of ELL.  **RESULTS:** The results showed seven main types of extramural learning activities (i.e., playing digital games, watching videos, reading, listening to audios, having technology-enhanced socialisation, having face-to-face socialisation, and writing compositions). People’s engagement in extramural language learning was overall high, especially listening to audios and playing digital games, mediated by the relationship between the difficulty of the activities and people’s target language proficiency levels, gender, and the interactive environment. Extramural language learning was overall effective for language development and enhancing affective states in language learning. The effectiveness may be influenced by the involvement of language inputs and outputs and the amount of engagement time.  **CONCLUSIONS:** Extramural language learning was overall effective and frequently engaged. Practitioners may encourage digital gameplay, emphasise formal language instruction, and create positive interactive environments in their implementation of extramural language learning.  **KEYWORDS:** extramural English; language learning; technology-enhanced language learning | 2 |
| **INTRODUCTION** | | | |
| Rationale | 3 | So far, the escalating empirical research of ELL has yet yielded incommensurate review studies in this field. To our best knowledge, few reviews have been conducted of the previous empirical studies on ELL. However, people might have engaged in many types of ELL activities and found them effective for various language learning aspects. Only when people's engagement and outcomes of all types of ELL activities have been examined can a comprehensive picture be attained of this language learning approach. Moreover, studies published after 2018 might have yet been reviewed. As research on ELL has continued to expand for the past years, an overview of the latest developments and trends in the field of ELL may be timely, suggesting implications and recommendations for future implementation and investigation. | 3-4 |
| Objectives | 4 | This research aims to conduct a review of previous empirical research on ELL with foci on the types of ELL activities and research findings concerning people’s engagement in ELL activities and the effectiveness of ELL. The following questions guide this review: What types of ELL activities were investigated? How frequently did people engage in ELL? Were ELL effective? What factors may influence people’s engagement in ELL? What factors may influence the effectiveness of ELL? | 4 |
| **METHODS** | | | |
| Protocol and registration | 5 | We conducted this review based on a three-step method: search, selection, and data analysis, following previous review studies in the field of language education. | 5 |
| Eligibility criteria | 6 | The articles were searched with “English” as the language, “all year” as time-span, and “article” for the required document type. To ensure the relevance of the reviewed articles, the articles were screened by the titles, abstracts, and full texts based on three inclusion criteria… First, the article should focus on language learning… Second, the article should focus on extramural learning… Third, the article should report an empirical study because this study focuses on the empirical evidence of the effectiveness of extramural language learning. | 5-6 |
| Information sources* | 7 | Our databases were Web of Science Core Collection (<https://login.webofknowledge.com>) and Scopus (<https://www.scopus.com>) that were frequently applied in previous review studies. | 6 |
| Search | 8 | 1. (extramural or out-of-school exposure) 2. (teach$ or learn$ or educat$) 3. (language or English or Chinese or Russian or Japanese or German or Spanish or Hindi or Portuguese) | N.A. |
| Selection of sources of evidence† | 9 | To begin the data analysis, the authors browsed the abstracts of the 33 articles to grab the big picture. Subsequently, we coded five articles together and discussed the coding scheme based on the research questions. Once an agreement had been reached, we analysed the remaining articles individually based on the coding scheme and compared our coding results. The satisfactory inter-rater reliability was reached (Pearson’s *r* = 0.96), and the remaining differences were resolved via discussion. | 8 |
| Data charting process‡ | 10 | The data-charting was developed by the first author who determined which variables to extract, charted the data, and updated the charting form during the iterative process. | N.A. |
| Data items | 11 | We abstracted data on types of extramural language learning activities (e.g., listening to audios, watching videos, reading, playing digital games, etc.), engagement of the extramural language learning activities, effects of the extramural language learning activities on language learning (e.g., positive, negative, neutral, mixed), target aspects of language learning (e.g., language development, affective states), and reasons for the effects of the extramural language learning activities. | N.A. |
| Critical appraisal of individual sources of evidence§ | 12 | Not appropriate. | N.A. |
| Synthesis of results | 13 | We summarised the types of extramural language learning activities investigated in the literature and grouped them according to the language skills involved therein. Also, we extracted data about people’s engagement in each extramural language learning activity. The effectiveness of extramural language learning activities was coded from the dimensions of target aspects of language learning, effects of the activities on language learning, and reasons reported in the literature for the effects. | N.A. |
| **RESULTS** | | | |
| Selection of sources of evidence | 14 | 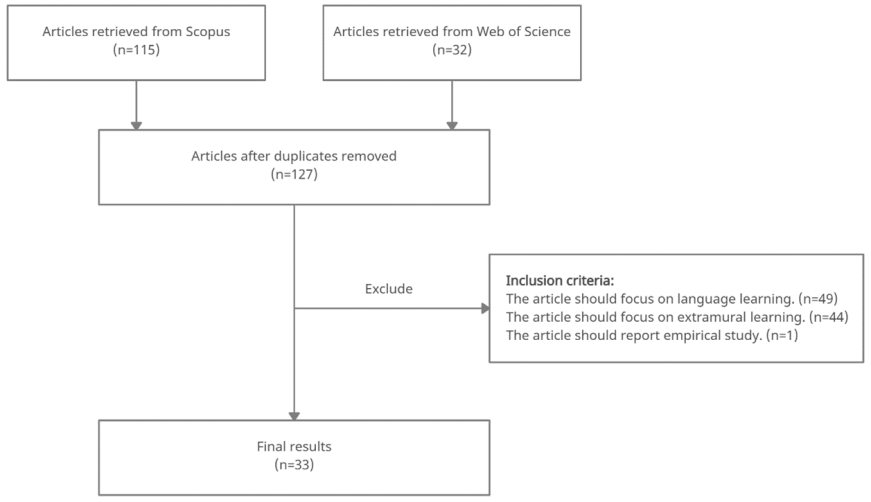 | 8 |
| Characteristics of sources of evidence | 15 | Thirty-three articles were finalised in the selection, as shown in Table 1.  S1 Appendix presents the summary of the results. | 6-8 |
| Critical appraisal within sources of evidence | 16 | Not appropriate. | N.A. |
| Results of individual sources of evidence | 17 | Please refer to S1 Appendix. | S1 Appendix |
| Synthesis of results | 18 | The main extramural language learning activities were playing digital games, watching videos, reading, listening to audios, having technology-enhanced socialisation, having face-to-face socialisation, and writing compositions. People’s engagement in extramural language learning was overall high, with listening to audios and playing digital games as the activities in which people engaged most frequently and reading as the one in which they engaged least frequently. Extramural language learning was overall effective in developing language and enhancing affective states in language learning. | 20 |
| **DISCUSSION** | | | |
| Summary of evidence | 19 | This study presents a review of 33 papers on extramural language learning, revealing the increasing application of this learning approach to language development. The main extramural language learning activities were playing digital games, watching videos, reading, listening to audios, having technology-enhanced socialisation, having face-to-face socialisation, and writing compositions. People’s engagement in ELL was overall high, with listening to audios and playing digital games as the activities in which people engaged most frequently and reading as the one in which they engaged least frequently. Extramural language learning was overall effective in developing language and enhancing affective states in language learning. Three factors may influence people's engagement in extramural language learning, specifically, the relationship between the difficulty of extramural language learning activities and people’s target language proficiency levels, gender, and the interactive environment of extramural language learning. The effectiveness of extramural language learning may be moderated by the language inputs and outputs involved therein and the amount of engagement time. | 19-20 |
| Limitations | 20 | This study is not without its limits. First, we searched for the reviewed articles in two databases, Scopus and Web of Science. Future studies may expand the body of data by including more databases, such as AHCI, ERIC, and Google Scholar, so as to present a more comprehensive picture of the field of ELL. Second, this study focused on the types of ELL activities, people’s engagement in ELL, and the effectiveness of ELL. Future studies may consider re-reviewing the literature from other aspects, such as the target language, the subjects' native languages and age, and sample sizes. | 22 |
| Conclusions | 21 | This review has identified people’s high engagement in ELL spanning diversified activities and suggested its apparent educational effectiveness for language development. Considering the great potential of this language learning approach, we expect further explorations of ELL from various perspectives, for example, learning behaviours in the process of ELL. | 22 |
| **FUNDING** | | | |
| Funding | 22 | The work described in this paper was supported by the One-off Special Fund from Central and Faculty Fund in Support of Research from 2019/20 to 2021/22 (MIT02/19-20) and the Research Cluster Fund (RG 78/2019-2020R) of The Education University of Hong Kong, Hong Kong, the Faculty Research Fund (DB21A9) and the HKIBS Research Program Grant Application (HCRG-201-002, 702024) of Lingnan University, Hong Kong, and the Open University of Hong Kong Research Grant (No. 2019/1.4). | 23 |

JBI = Joanna Briggs Institute; PRISMA-ScR = Preferred Reporting Items for Systematic reviews and Meta-Analyses extension for Scoping Reviews.

* Where *sources of evidence* (see second footnote) are compiled from, such as bibliographic databases, social media platforms, and Web sites.

† A more inclusive/heterogeneous term used to account for the different types of evidence or data sources (e.g., quantitative and/or qualitative research, expert opinion, and policy documents) that may be eligible in a scoping review as opposed to only studies. This is not to be confused with *information sources* (see first footnote).

‡ The frameworks by Arksey and O’Malley (6) and Levac and colleagues (7) and the JBI guidance (4, 5) refer to the process of data extraction in a scoping review as data charting*.*

§ The process of systematically examining research evidence to assess its validity, results, and relevance before using it to inform a decision. This term is used for items 12 and 19 instead of "risk of bias" (which is more applicable to systematic reviews of interventions) to include and acknowledge the various sources of evidence that may be used in a scoping review (e.g., quantitative and/or qualitative research, expert opinion, and policy document).

*From:* Tricco AC, Lillie E, Zarin W, O'Brien KK, Colquhoun H, Levac D, et al. PRISMA Extension for Scoping Reviews (PRISMAScR): Checklist and Explanation. Ann Intern Med. 2018;169:467–473. [doi: 10.7326/M18-0850](http://annals.org/aim/fullarticle/2700389/prisma-extension-scoping-reviews-prisma-scr-checklist-explanation).
